# Prognostic Implication of Patient Age in H3K27M-Mutant Midline Gliomas

**DOI:** 10.3389/fonc.2022.858148

**Published:** 2022-03-18

**Authors:** Huy Gia Vuong, Tam N. M. Ngo, Hieu Trong Le, Andrew Jea, Maya Hrachova, James Battiste, Rene McNall-Knapp, Ian F. Dunn

**Affiliations:** ^1^ Department of Neurosurgery, The University of Oklahoma Health Sciences Center, Oklahoma University, Oklahoma City, OK, United States; ^2^ Faculty of Medicine, Pham Ngoc Thach University of Medicine, Ho Chi Minh City, Vietnam; ^3^ Department of Pathology, University of Medicine and Pharmacy at Ho Chi Minh City, Ho Chi Minh City, Vietnam; ^4^ Department of Neurosurgery, Division of Pediatric Neurosurgery, Oklahoma Children’s Hospital, The University of Oklahoma Health Sciences Center, Oklahoma University, Oklahoma City, OK, United States; ^5^ Department of Pediatrics, The University of Oklahoma Health Sciences Center, Oklahoma University, Oklahoma City, OK, United States

**Keywords:** H3K27M, midline glioma, H3F3A, HIST1H3B/C, pediatric, adult, overall survival, progression-free survival

## Abstract

**Introduction:**

Pediatric and adult *H3*K27M-mutant midline gliomas have variable clinical presentations, prognoses, and molecular backgrounds. In this study, we integrated data from published studies to investigate the differences between these two groups.

**Methods:**

PubMed and Web of Science were searched for potential data. Studies were included if they had available individual participant data on patients age of *H3*K27M-mutant midline gliomas. For time-to-event analyses, Kaplan-Meier analysis and Cox regression models were carried out; corresponding hazard ratios (HR) and 95% confidence intervals (CI) were computed to analyze the impact of age and clinical covariates on progression-free survival (PFS) and overall survival (OS).

**Results:**

We included 43 studies comprising 272 adults and 657 pediatric midline gliomas with *H3*K27M mutation for analyses. In adults, there was a male predilection whereas females were slightly more common than males in the pediatric group. Spinal cord tumors were more frequent in adults. The prevalence of *H3.1* K27M mutation was significantly higher in the pediatric cohort. Compared to adult patients, pediatric *H3*K27M-mutant midline gliomas exhibited more aggressive features including higher rates of pathologic features of high-grade tumors and Ki67 proliferation index, and had a shorter PFS and OS. Genetically, *ACVR1* mutations were more common whereas *MGMT* methylation, *FGFR1*, and *NF1* mutations were less prevalent in the pediatric cohort.

**Conclusion:**

Pediatric *H3*K27M-mutant midline gliomas were demographically, clinically, and molecularly distinct from adult patients, highlighting an opportunity to refine the risk stratification for these neoplasms.

## Introduction

*H3*K27M-mutant glial tumors arise predominantly in midline structures such as the thalamus, brainstem, and spinal cord (1, 2). The *H3*K27M-mutant diffuse midline glioma is a relatively newly described entity, debuting in the revised 2016 World Health Organization classification of tumors of the central nervous system. These tumors are more commonly seen in pediatric patients and are associated with a poor prognosis of typically less than one year because of their infiltrative nature and difficulty in achieving complete surgical resection (3–6). *H3* mutations could occur either on the *HIST1H3B/C* (*H3.1*), *HIST2H3C* (*H3.2*), or *H3F3A* (*H3.3*) genes with a lysine to methionine amino acid substitution at codon 27 (K27M) and are mutually exclusive with isocitrate dehydrogenase 1/2 (*IDH1/2)* mutations (7).

Despite aggressive therapeutic approaches and advances in chemoradiotherapy and targeted therapy regimens, there has been no survival improvement for patients with *H3*K27M mutations over the recent years (3, 8). Published studies have shown several differences in clinicopathological parameters between pediatric and adult patients with *H3*K27M-mutant gliomas (9, 10), but no significant differences in overall survival (11, 12). In this study, individual patient data from published studies were integrated to investigate the clinical and prognostic differences between pediatric and adult *H3*K27M-mutant midline gliomas.

## Methods

### Search Term and Literature Search

We accessed PubMed and Web of Science to search for relevant articles from inception to July 2021 using the following search term: Glioma AND (H3K27M OR H3-K27M OR H3 K27M OR H3F3A OR HIST1H3B OR HIST1H3C). The study protocol was strictly adherent to the recommendations of the Preferred Reporting Items for Systematic Review and Meta-Analysis (PRISMA) statement (13).

### Selection Criteria and Abstract Screening

Results from the two electronic databases were imported into EndNote (Clarivate, PA, USA) and duplicates were subsequently removed. Next, two reviewers (HGV and TNMN) independently screened the title and abstract of the articles using the following inclusion criteria: (i) studies providing individual participant data (IPD) of *H3*K27M-mutant gliomas and (ii) studies with patient age available. We excluded studies if they were: (i) not relevant to inclusion criteria; (ii) case reports; (iii) reviews, theses, or books; (iv) conference or proceeding papers; or (v) studies with duplicated populations.

### Full-Text Screening and Data Extraction

Two reviewers (HGV and TNMN) independently reviewed the full text of potential studies and extracted data into a standardized worksheet. We also carefully reviewed the reference list of the included studies to find additional papers. The following IPD were extracted from the articles: authors, institution, country, year of publication, study period, patient identification number, *H3* genotypes, detection methods, demographic information, tumor size, tumor location, histology grades, Ki67 index, treatments, PFS time, PFS status, OS time, OS status, and genetic alterations of *H3*K27M-mutant gliomas. Subsequently, we removed cases with missing data of patient age/tumor location or cases from non-midline locations.

### Statistical Analyses

Patients were divided into pediatric (patients of 18 or less than 18 years of age) and adult groups (patients of more than 18 years of age). To avoid duplicated data between studies from the same institutions, we selected the study with the largest population. Categorical and continuous variables of pediatric and adult cohorts were compared utilizing Chi-square, Fisher’s exact test, and Wilcoxon rank-sum test, if applicable. Kaplan-Meier analysis and Cox proportional hazards model were conducted to assess the impact of various clinical parameters on survival of *H3*K27M-mutant gliomas. The deviance residuals and the dfbeta values were used to examine influential observations. Hazard ratios (HR) are presented as mean and 95% confidence interval (CI). A two-sided *p*-value of < 0.05 was considered statistically significant. The statistical analyses were performed using the R software, version 3.6.1 (The R Foundation, Vienna, Austria).

## Results

**Figure 1:**
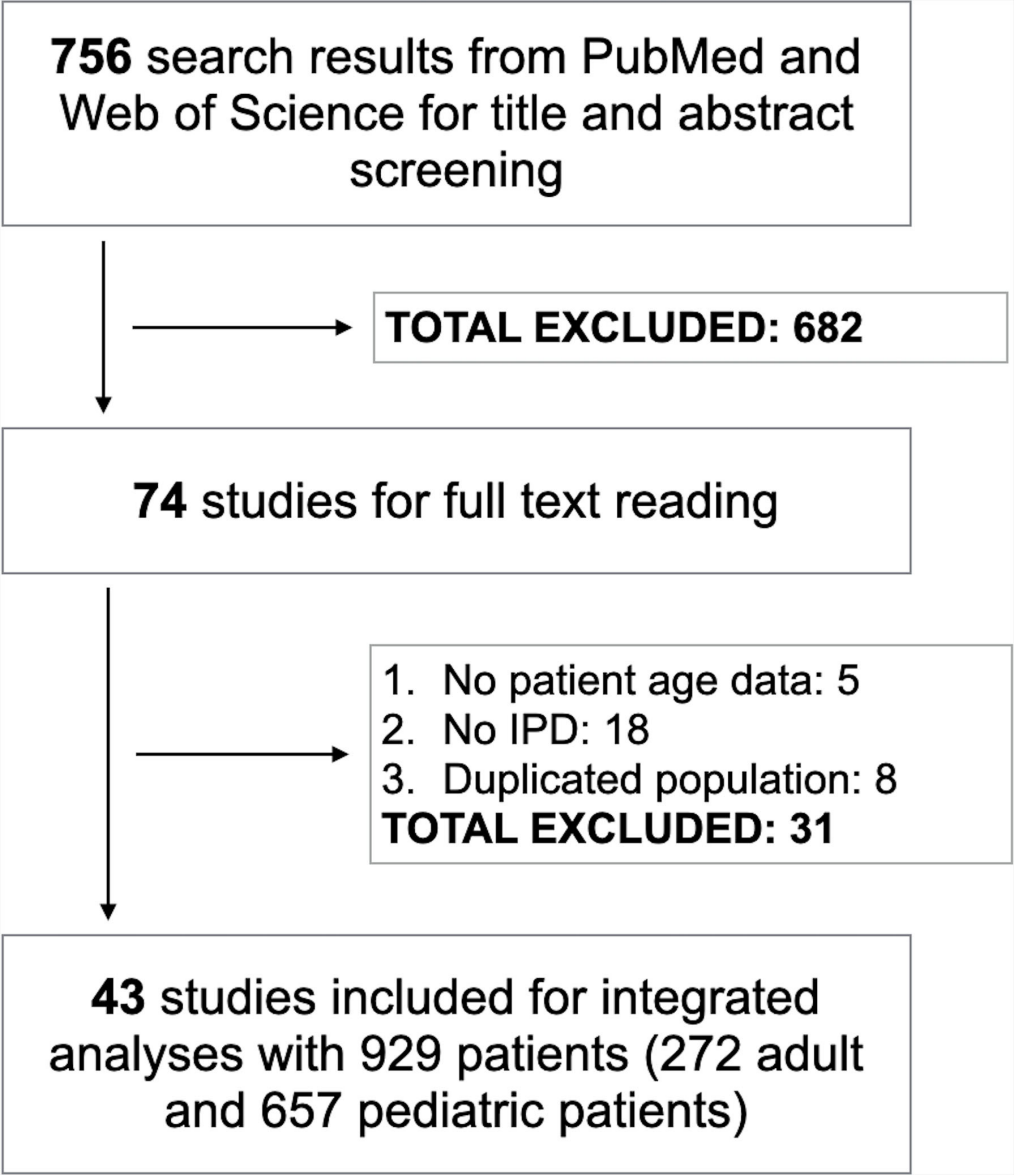
Study flowchart. IPD, individual participant data.

For the title and abstract screening, we identified 756 articles, and 74 of them were selected for full-text review. After reading full text, we included 43 studies comprised of 929 *H3*K27M-mutant midline gliomas for integrated analyses (**Figure 1**) (2, 4, 7, 8, 10–12, 14–44). The characteristics of all included studies are shown in **Table S1**. The median age of patients was 11.0 years (range, 1-82). The majority of tumors were found intracranially with brainstem or thalamus being the most frequent sites. The median OS was 11.3 months with 80% of patients expired at last follow-up. Stratified by age, 272 and 657 patients were separated into adult and pediatric groups, respectively.

### The Differences Between Pediatric and Adult Midline Gliomas With *H3*K27M Mutations

**Table 1** presents the clinicopathological and therapeutic covariates of adult and pediatric *H3*K27M-mutant midline gliomas. In comparison to the adult counterpart, the K27M mutation in the *H3.1* gene was more commonly identified in pediatric patients (p < 0.001). There was a male predilection in adult *H3*K27M-mutant gliomas whereas females were more common in the pediatric group (p < 0.001). In the pediatric population, *H3*K27M-mutant tumors were mostly seen in the brainstem whereas a higher proportion of thalamic and spinal lesions were identified in adult patients (p< 0.001). Adult tumors had a lower Ki67 index compared to the pediatric group (p < 0.001).

**Table 1:** The clinicopathological parameters of pediatric versus adult *H3*K27M-mutant midline gliomas.

Clinicopathological parameters	Adult (n = 272)	Pediatric (n = 657)	All cases (n = 929)	p-value
*H3* Genotype (%)				< 0.001
*H3.*1 & *H3.2*	5 (3.3)	97 (20.2)	102 (16.2)	
* H3.3*	146 (96.7)	383 (79.8)	529 (83.8)	
Gender (%)				<0.001
Female	98 (38.7)	333 (52.2)	323 (48.1)	
Male	155 (61.3)	305 (47.8)	348 (51.9)	
Tumor location (%)				<0.001
Brainstem	63 (23.2)	404 (69.5)	467 (54.7)	
Thalamus	104 (38.2)	124 (21.3)	228 (26.7)	
Spinal cord	61 (22.4)	30 (5.2)	91 (10.7)	
Other midline locations	44 (16.2)	23 (4.0)	67 (7.9)	
Histology WHO grade (%)				0.031
High grade	175 (80.6)	492 (86.8)	667 (85.1)	
Low grade	42 (19.4)	75 (13.2)	117 (14.9)	
Ki67 index (%)				<0.001
Mean (SD)	20.1 (19.1)	29.1 (16.4)	24.8 (18.3)	
Median [IQR]	13.5 [6.5; 30.0]	30.0 [20.0; 40.0]	20.0 [10.0; 25.0]	
Surgery (%)				<0.001
GTR	12 (7.7)	29 (13.2)	41 (10.9)	
PR	30 (19.2)	18 (8.22)	48 (12.8)	
STR	27 (17.3)	90 (41.1)	117 (31.2)	
Biopsy	87 (55.8)	82 (37.4)	169 (45.1)	
Radiotherapy (%)				<0.001
No	18 (15.4)	9 (3.70)	27 (7.50)	
Yes	99 (84.6)	234 (96.3)	333 (92.5)	
Chemotherapy (%)				0.564
No	14 (11.9)	45 (14.0)	59 (13.4)	
Yes	104 (88.1)	277 (86.0)	381 (86.6)	

GTR, gross total resection; IQR, interquartile range; PR, partial resection; SD, standard deviation; STR, subtotal resection.

**Figure 2:**
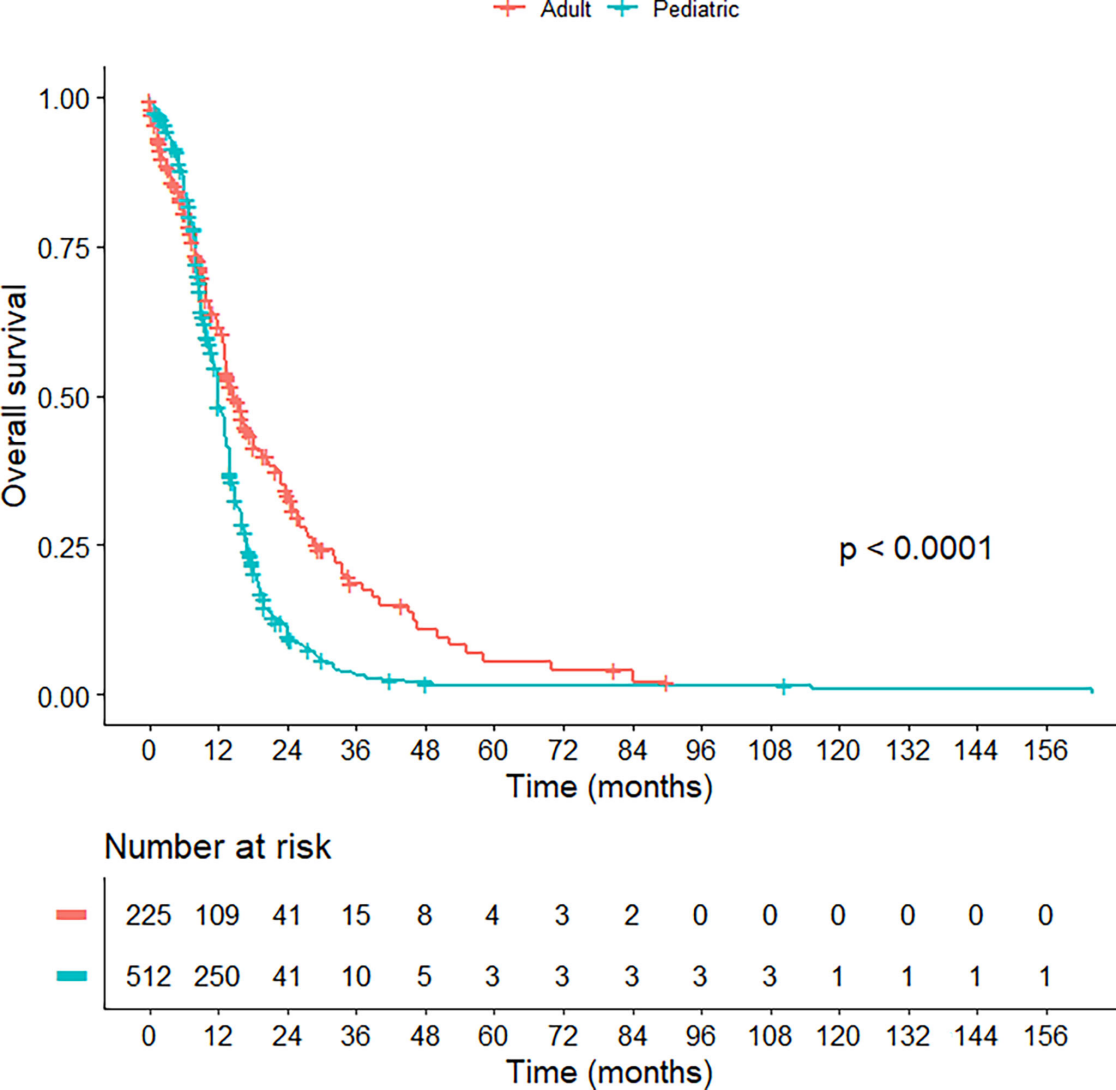
Kaplan-Meier curve illustrating the overall survival of pediatric versus adult *H3*K27M-mutant midline gliomas.

**Figure 3:**
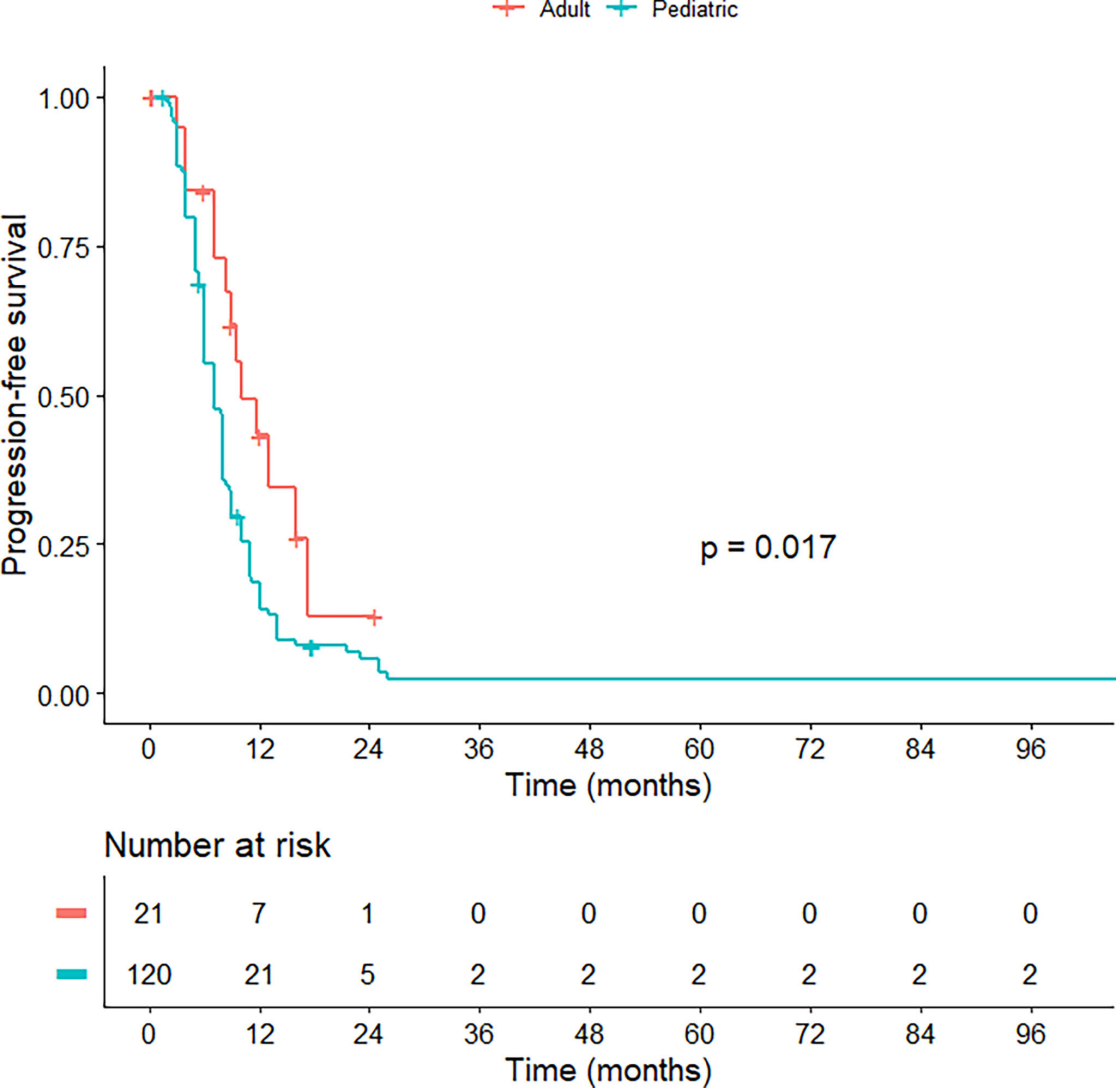
Kaplan-Meier curve illustrating the progression-free survival of pediatric versus adult *H3*K27M-mutant midline gliomas.

Regarding treatment modalities, pediatric *H3*K27M-mutant gliomas were associated with higher rates of gross total/subtotal removal and radiotherapy administration (p < 0.001). In comparison to the adult group, pediatric patients had a worse OS (median OS of 13.0 vs 14.7 months; HR = 1.630; 95% CI = 1.347-1.973; p < 0.001) (**Figure 2**) and PFS (median PFS of 7.0 vs 9.0 months; HR = 2.015; 95% CI = 1.132-3.587; p = 0.017) (**Figure 3**). When pediatric patients were further stratified into different subgroups of infants (0 < age ≤ 3), young children (3 < age ≤ 10), and adolescents (10 < age ≤ 18), there was a trend to better OS of infants (median OS of 14.5 months) as compared to young children (median OS of 11.3 months; p = 0.063) and adolescents (median OS of 10.8 months; p = 0.113) (**Figure S1**). We did not see any differences in survival patterns between young adults (19 < age ≤ 45), older adults (45 < age ≤ 60), and elderly (age > 60) (p = 0.67) (**Figure S2**).

Among intracranial tumors, OS of pediatric *H3*K27M-mutant gliomas remained significantly shorter (HR = 1.430; 95% CI = 1.162-1.759; p < 0.001) (**Figure S3**). We also observed a similar OS trend for spinal *H3*K27M-mutant midline gliomas, but the difference did not reach statistical significance (HR = 2.035; 95% CI = 0.914-4.534; p = 0.082) (**Figure S4**). Diffuse intrinsic pontine gliomas (DIPG) were seen in 33.6% and 5.5% of pediatric and adult H3K27M-mutant midline gliomas. We still observed a significant difference in overall survival between pediatric and adult patients after excluding them from the analysis (**Figure S5**). Because of missing data, we could not stratify PFS analysis into subgroups.

### Prognostic Factors for OS of *H3*K27M-Mutant Midline Gliomas

In a multivariate Cox regression model for OS of *H3*K27M-mutant midline gliomas adjusted for age, gender, *H3* genotype, tumor location, the extent of surgical resection, radiation, and chemotherapy; advanced age and radiotherapy administration were positive prognostic factors for overall survival. Other parameters were not associated with patient survival (**Table 2**).

**Table 2:** Multivariate Cox regression analysis for overall survival of *H3*K27M-mutant midline gliomas.

Clinical parameters		Hazard ratio (95% CI)	p-value
Age	Per year increase	0.966 (0.948-0.985)	<0.001
Gender	Female	Reference	
	Male	0.954 (0.624-1.457)	0.826
*H3* Genotype	*H3.1* & *H3.2*	Reference	
	*H3.3*	1.811 (0.682-4.814)	0.231
Location	Intracranial	Reference	
	Spinal cord	1.488 (0.704-3.143)	0.298
Surgery	Biopsy	Reference	
	Resection	0.920 (0.533-1.585)	0.763
Radiotherapy	No	Reference	
	Yes	0.355 (0.171-0.738)	0.005
Chemotherapy	No	Reference	
	Yes	0.885 (0.485-1.617)	0.691

CI, confidence interval.

### Genetic Alterations of Pediatric Versus Adult *H3*K27M-Mutant Midline Gliomas

We observed a significantly higher prevalence of *MGMT* methylation, *NF1*, and *FGFR1* mutations in adults in comparison to pediatric patients whereas *ACVR1* mutations were solely detected in children. The frequencies of *EGFR* and *PDGFRA* amplifications*, ATRX, BRAF, PDGFRA, PIK3CA, PPM1D, TERT* promoter, and *TP53* mutations were not statistically different between these two groups (**Table 3**).

**Table 3:** The genetic alterations of pediatric versus adult *H3*K27M-mutant midline gliomas.

Genetic markers	Adult (%)	Pediatric (%)	All cases (%)	p-value
*ACVR1* mutation	0/22 (0)	60/315 (19.0)	60/337 (17.8)	0.020
ATRX loss	13/49 (26.5)	21/78 (26.9)	34/127 (26.8)	0.961
*ATRX* mutation	16/81 (19.8)	41/275 (14.9)	57/356 (16.0)	0.296
*BRAF* mutation	1/97 (1.0)	11/291 (3.8)	12/388 (3.1)	0.308
*EGFR* amplification	1/60 (1.7)	6/83 (7.2)	7/143 (4.9)	0.239
*FGFR1* mutation	10/43 (23.3)	11/124 (8.9)	21/167 (12.6)	0.014
*MGMT* methylation	17/114 (14.9)	8/131 (6.1)	25/245 (10.2)	0.023
*NF1* mutation	7/29 (24.1)	14/202 (6.9)	21/231 (9.1)	0.008
*PDGFRA* amplification	0/2 (0)	43/218 (19.7)	43/220 (19.5)	0.996
*PDGFRA* mutation	6/32 (18.8)	14/161 (8.7)	20/193 (10.4)	0.110
*PIK3CA* mutation	2/20 (10.0)	37/208 (17.8)	39/228 (17.1)	0.377
*PPM1D* mutation	2/17 (11.8)	24/185 (13.0)	26/202 (12.9)	1.000
*TERT* mutation	5/76 (6.6)	2/65 (3.1)	7/141 (5.0)	0.452
*TP53* mutation	48/83 (57.8)	173/335 (51.6)	221/418 (52.9)	0.312

## Discussion

Diffuse midline gliomas are mostly high-grade tumors and have a lethal outcome. These neoplasms can occur at any age from infants to elderly patients but are most commonly found in children (35, 45, 46). About 80-90% of these tumors harbor mutations in *H3* genes, most of which are *H3*K27M genotypes (45). *H3*K27M-mutant midline gliomas are associated with a worse outcome compared to *H3-*wild-type tumors (4). Because midline gliomas with *H3*K27M mutations are clinically different from H3K27M-mutated cortical high-grade gliomas (47), we only focused on midline tumors to avoid the risk of bias. Given its rarity in adults, it is challenging to investigate the differences in clinical manifestations and prognosis between pediatric and adult groups (11). In this study, we conducted an integrated analysis of more than 900 *H3*K27M-mutated midline gliomas to examine the clinicopathological parameters and prognosis between adults versus pediatrics. Our results showed that pediatric tumors were not only clinically and prognostically different but also had distinct molecular profiles as compared to the adult group.

In this study of *H3*K27M-mutant midline tumors, young age portended an adverse prognosis with shorter PFS and OS as compared with adult patients. While earlier reports suggested that OS of pediatric and adult *H3*K27M-mutant gliomas are not different (8.9 vs 9.3 months, respectively), only a small number of patients were compared (11). Schulte *et al.* (48) reported a higher survival rate of adult H3K27M midline gliomas compared to children in published case series and hypothesized that adults might have a better survival. Notably, our results revealed that pediatric patients had a higher rate of tumor resection and radiation administration than adult patients, substantiating that pediatric *H3*K27M-mutant midline gliomas carry worse outcome despite more aggressive treatment attempts than in adult patients. Our findings showed that tumors in pediatric patients had more aggressive pathologic features including a significantly advanced histological grade and Ki67 proliferation index, which may explain the poor survival of the pediatric cohort. Hoffmann *et al.* reported that pediatric DIPG with age of less than 3 and more than 10 years were associated with longer-term survival compared to those aged 3-10 (49). In this study, although the p-value of the log-rank test demonstrated significant differences between these three age subgroups, pairwise comparison showed that the differences between infants vs young children and infants vs adolescents did not reach statistical significance.

While earlier studies reported a high frequency of *H3* K27M mutation in both adult and pediatric high-grade spinal gliomas (50), we found a significantly higher frequency of adult *H3* K27M spinal cord tumors as compared to the pediatric population. These results were consistent with published population-level data on high-grade gliomas and glioblastomas of the spinal cord that these neoplasms are most commonly seen in adults (51, 52), as opposed to DIPGs or brainstem gliomas which are more likely to occur in the pediatric population (3, 8). Several reports indicated the aggressiveness of *H3*K27M-mutant diffuse midline gliomas is independent of their anatomical locations (1, 53). However, other published data reported that patient survival may be influenced by different anatomical locations (42, 54). When we stratified cases into subgroups of intracranial and spinal tumors, we still observed the same result for pediatric intracranial tumors and a similar trend for pediatric spinal gliomas, possibly due to the relatively small sample size of spinal cord gliomas. These results confirmed the uniformly short survival of *H3*K27M midline gliomas in the pediatric population, regardless of anatomical location.

Compared to pediatric tumors, adult *H3*K27M-mutant midline gliomas were also genetically different. We found that *ACVR1* mutations were exclusively seen in pediatric patients, particularly in children less than six years of age. On the other hand, the incidence of *FGFR1, NF1* mutations, and *MGMT* methylation were more significantly prevalent in adult patients. The lower frequency of *MGMT* hypermethylation in pediatric patients may contribute to the poor response to temozolomide and a worse PFS/OS as compared to adult cohort. *FGFR1* mutations are associated with prolonged survival in *H3*K27M-mutant gliomas (55), which may influence the more favorable outcome in adult patients. Other genetic alterations such as *EGFR* alterations, *TERT* promoter mutations, and mutations in the tumor suppressor *PTEN* are less likely to exist in high-grade gliomas in children (56, 57). However, we observed an insignificant difference in the prevalence of these genetic markers between pediatric and adult *H3*K27M-mutated midline gliomas. Hopefully, these observations may help understand the molecular profile of midline gliomas as treatment.

This study is the first study to demonstrate the differences in demographics, clinical manifestations, prognoses, as well as molecular backgrounds of pediatric versus adult *H3*K27M-mutant midline gliomas. Because of the rarity of *H3*K27M-mutant midline gliomas in adult patients, it is challenging to observe the significant differences between pediatric and adult cohorts in institutional studies (11). Our results are of clinical interest as they may aid in refining natural history expectations and in tailoring therapeutic approaches. However, this study has certain limitations. First, we could not avoid selection bias originating from the included datasets because most of them were retrospective studies. In the multivariate Cox regression model adjusted for multiple clinical covariates, the prognostic implication of age remained significant, validating the independent role of this parameter in the risk stratification of *H3*K27M-mutant midline gliomas. Additionally, there are important clinical factors that we could not incorporate into the multivariate analysis due to missing data, such as the Karnofsky Performance Scale, tumor size, and disease stage. Pediatric patients may have limited self-care ability resulting in lower KPS and advanced disease stages compared with adults. Future prospective studies are needed to confirm the results of this study. Another bias could occur due to differences in treatment protocols across included institutions. Finally, although we strictly screened the included patients using patient identification code, demographic information, and clinical data to avoid duplicating patients, there are still possibilities of overlap among the dataset which may affect the analyses.

In conclusion, the present study demonstrated that pediatric *H3*K27M-mutant midline gliomas are demographically, clinically, prognostically distinct from adult tumors. H3K27- mutant midline gliomas in pediatric patients were associated with a significantly shorter PFS and OS. Additionally, the underlying molecular backgrounds of these two cohorts were also different. Our results may improve our biological understanding of H3K27M-mutant midline gliomas.

## Data Availability Statement

The original contributions presented in the study are included in the article/**Supplementary Material**. Further inquiries can be directed to the corresponding author.

## Author Contributions

HV, conceptualization, data curation, formal analysis, investigation, methodology, project administration, software, validation, writing original, review, and editing. TN, HL, AJ, MH, JB, and RM-K, data curation, formal analysis, investigation, methodology, review, and editing. IFD, data curation, formal analysis, investigation, methodology, project administration, validation, review, editing, and supervision. All authors contributed to the article and approved the submitted version.

## Conflict of Interest

The authors declare that the research was conducted in the absence of any commercial or financial relationships that could be construed as a potential conflict of interest.

## Publisher’s Note

All claims expressed in this article are solely those of the authors and do not necessarily represent those of their affiliated organizations, or those of the publisher, the editors and the reviewers. Any product that may be evaluated in this article, or claim that may be made by its manufacturer, is not guaranteed or endorsed by the publisher.

## Abbreviations

CI, confidence interval; H3.1, HIST1H3B/C; H3.2, HIST2H3C; H3.3, H3F3A; HR, hazard ratio; IDH, isocitrate dehydrogenase; IPD, individual participant data; OS, overall survival; PFS, progression-free survival.
