# Supplementary material for: Prognostic Implication of Patient Age in H3K27M-Mutant Midline Gliomas
Source: Front Oncol. 2022 Mar 18;12:858148. doi: 10.3389/fonc.2022.858148 (PMC8971724; doi:10.3389/fonc.2022.858148)

**Prognostic implication of patient age in H3K27M-mutant midline gliomas**

Vuong et al.

(Supplementary materials)

Table S1. The characteristics of 43 included studies

| Study | Country | No. of patients | *H3* mutation  detection methods | Mean age  (SD) | Median follow-up (months) |
| --- | --- | --- | --- | --- | --- |
| Aihara 2014 | Japan | 10 | Sanger | 34.2 (9.76) | 10.2 |
| Alvi 2019 | USA | 6 | Sanger | 30.5 (9.83) | 10.5 |
| Bruzek 2020 | USA | 9 | Sanger | 10.0 (4.66) | 14.0 |
| Buczkowicz 2014 | Canada | 23 | NGS | 6.3 (2.70) | 12.0 |
| Castel 2015 | France | 79 | NGS | 7.5 (3.41-3.39) | 10.4 |
| Chiba 2020 | Japan | 4 | IHC | 13.1 (3.85) | N/A |
| Crotty 2020 | United States | 10 | NGS | 8.6 (5.32) | N/A |
| Daoud 2018 | USA | 6 | IHC | 38.5 (11.04) | 7.8 |
| Dono 2020 | USA | 3 | NGS | 31.3 (7.64) | 16.7 |
| Dorfer 2020 | Austria | 14 | Sanger | 8.9 (4.04) | 13.8 |
| Ebrahimi 2019 | Germany | 40 | IHC/Pyroseq | 33.7 (20.72) | N/A |
| Eschbacher 2021 | USA | 6 | NGS/IHC | 37.2 (17.13) | 14.9 |
| Fontebasso 2014 | Canada | 37 | WES | 8.9 (4.27) | N/A |
| Fukami 2018 | Japan | 11 | Sanger | 40.2 (21.27) | 12.0 |
| Garibotto 2020 | Italy | 12 | NGS | 8.7 (3.89) | 9.5 |
| Gessi 2015 | Germany | 17 | IHC/Pyroseq | 24.8 (17.73) | N/A |
| Giagnacovo 2020 | Italy | 19 | Sanger | 8.6 (2.93) | 11.0 |
| Gojo 2020 | Austria & Czech | 18 | IHC | 8.3 (4.56) | 17.2 |
| Grasso 2015 | USA | 25 | NGS | 7.2 (4.36) | 10.0 |
| Hoffman 2016 | USA | 7 | IHC | 8.3 (7.36) | 13.4 |
| Karlowee 2019 | Japan | 12 | IHC | 27.9 (15.34) | 14.6 |
| Kleinschmidt-DeMasters 2018 | USA | 28 | Sanger | 28.7 (25.53) | 8.9 |
| Korshunov 2015 | Russia | 68 | Sanger | 10.1 (4.29) | 12.0 |
| Liu 2019 | China | 12 | WES | 29.1 (11.79) | 8.0 |
| Mackay 2017 | Multi | 17 | NGS/Sanger | 9.3 (3.97) | 9.0 |
| Mackay 2018 | Multi | 22 | Sanger | 11.9 (3.44) | 12.0 |
| Meyronet 2017 | France | 21 | NGS | 35.3 (16.95) | 10.0 |
| Mueller 2019 | USA | 13 | Sanger | 7.4 (3.25) | 13.1 |
| Nomura 2017 | Japan | 3 | Sanger | 41.7 (22.81) | 11.3 |
| Pan 2019 | China | 34 | Digital droplet PCR | 19.7 (16.90) | 9.4 |
| Panditharatna 2018 | USA | 46 | Digital droplet PCR | 8.6 (5.15) | N/A |
| Picca 2018 | France | 37 | NGS | 36.7 (15.39) | 16.2 |
| Reinhardt 2019 | Multi | 7 | IHC | 32.4 (15.86) | 6.5 |
| Ryall 2016 | Canada | 16 | IHC/Pyroseq | 10.4 (3.23) | 12.3 |
| Schwartzentruber 2012 | Multi | 4 | NGS | 11.3 (1.53) | 7.0 |
| Sievers 2021 | Germany | 8 | NGS | 5.0 (1.10) | 10.0 |
| Sturm 2012 | Multi | 12 | Sanger | 10.7 (4.52) | 12.5 |
| Taylor 2014 | Multi | 35 | Sanger | 7.2 (2.39) | 13.1 |
| Wang 2018 | China | 59 | WGS/WES | 27.5 (17.90) | 6.0 |
| Wang 2020 | China | 43 | Sanger | 35.4 (19.39) | 8.0 |
| Wu 2014 | USA | 52 | WGS/WES | 7.9 (4.31) | 10.2 |
| Yi 2019 | Korea | 20 | Sanger | 35.5 (13.93) | 25.5 |
| Zhou 2021 | China | 4 | Multiplexed PCR | N/A | 7.4 |

Abbreviations: IHC, immunohistochemistry; N/A, not available; NGS, next-generation sequencing; Pyroseq, pyrosequencing; SD, standard deviation; WES, whole-exome sequencing; WGS, whole-genome sequencing

Figure S1. Kaplan-Meier curve illustrating the overall survival of pediatric subgroups. Pairwise comparisons: infants vs young children, p = 0.063; infants vs adolescents, p = 0.113; young children vs adolescents (p = 0.136)

Figure S2. Kaplan-Meier curve illustrating the overall survival of adult subgroups

Figure S3. Kaplan-Meier curve illustrating the overall survival of pediatric versus adult patients with intracranial *H3*K27M-mutant midline gliomas

Figure S4. Kaplan-Meier curve illustrating the overall survival of pediatric versus adult patients with spinal cord *H3*K27M-mutant gliomas

Figure S5. Kaplan Meier analysis for overall survival of pediatric and adult H3K27M-mutant diffuse midline gliomas after removing cases with diffuse intrinsic pontine glioma


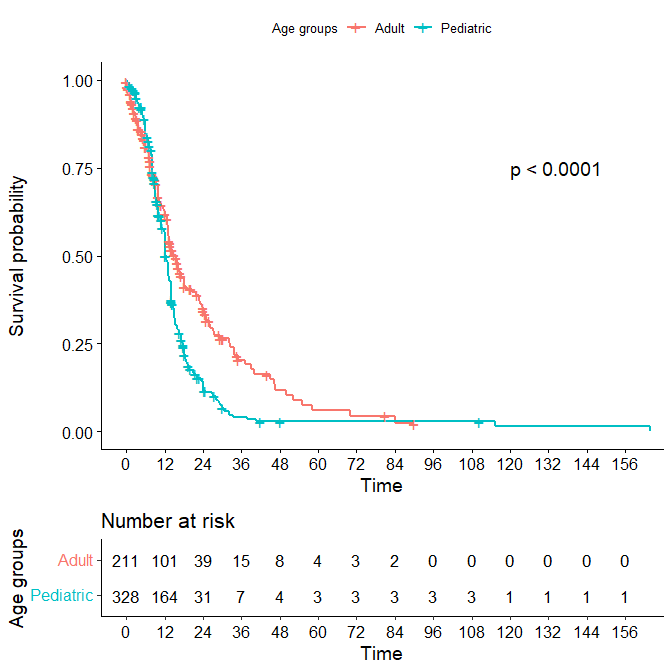

Supplement: Supplementary file 1 [file DataSheet_1.docx]
